# Functional regions of HpaXm as elicitors with specific heat tolerance induce the hypersensitive response or plant growth promotion in nonhost plants

**DOI:** 10.1371/journal.pone.0188788

**Published:** 2018-01-03

**Authors:** Yue Liu, Xiaoyun Zhou, Wenbo Liu, Xiaohui Xiong, Chuyang Lv, Xiang Zhou, Weiguo Miao

**Affiliations:** 1 Institute of Tropical Agriculture and Forestry, Hainan University, Haikou, Hainan Province, China; 2 Hainan Key Laboratory for Sustainable Utilization of Tropical Bioresource, Haikou, Hainan Province, China; Texas Tech University, UNITED STATES

## Abstract

HpaXm produced by the cotton leaf blight bacterium *Xanthomonas citri* subsp. *malvacearum* is a novel harpin elicitor of the induced hypersensitive response (HR) in tobacco. We investigated whether fragments of HpaXm, compared with fragments of Hpa1Xoo, are sufficient for HR or plant growth promotion (PGP) elicitation using four synthetic peptides (HpaXm_35-51_, HpaXm_10-39_, Hpa1Xoo_36-52_ and Hpa1Xoo_10-40_). We also heated the fragments to determine the heat tolerance of the functional fragments. HpaXm_35-51_ and Hpa1Xoo_36-52_ induced hypersensitive response (HR). Bursts of reactive oxygen intermediates (ROI) induced by HpaXm_35-51_ and Hpa1Xoo_36-52_ were earlier and stronger than those induced by HpaXm and Hpa1Xoo. In plants treated with HpaXm_35-51_ or Hpa1Xoo_36-52_, the expression of the HR marker genes *Hin1 and Hsr203J* and the active oxygen metabolism related gene *AOX* were significantly upregulated. These findings suggest that the predicted α-helical structures of the HpaXm_35-51_ and Hpa1Xoo_36-52_ fragments are crucial for HR. PGP result by soaking seeds in unheated/heated HpaXm_10-39_ or Hpa1Xoo_10-40_ solution prior to transfer, which obviously enhances root growth and the aerial parts of plants. The PGP related gene *NtEXP6* was greatly enhanced when plants were sprayed with a solution of HpaXm_10-39_ or Hpa1Xoo_10-40_; heated fragment treatments induced higher levels of *NtEXP6* expression than unheated HpaXm fragments. In addition, HR marker genes induced by the heated fragments had lower expression levels than when induced with unheated HpaXm fragments. Moreover, the expression levels of HR marker genes and PGP related genes induced by treatment with Hpa1Xoo fragments before or after heating were the opposite of those induced by HpaXm fragments. Different functional fragments of harpin and different harpins with the same functional region have different degrees of heat tolerance. Therefore, the heat resistance of harpin is conservative, but the degree of heat tolerance of the functional fragments is specific.

## Introduction

Harpins, encoded by *hrp* (hypersensitive response and pathogenicity) genes, are secreted by Gram-negative bacteria during interactions with host plants to cause diseases [1]. Since the first harpin of pathogenic origin, HrpN of *Erwinia amylovora* [2], was reported in 1992 as a cell-free elicitor of the hypersensitive response (HR), several other harpins have been characterized [1]. Interestingly, in addition to HR activity, diverse beneficial biological activities induced by harpins have been determined. For instance, the harpin-encoding gene *hrpN* of *E*. *amylovora* induces disease resistance through the systemic acquired resistance (SAR) pathway in *Arabidopsis* [3]; HrpN induces drought tolerance in *Arabidopsis* mediated by ABI2-dependent abscisic acid signaling [4]; Hpa1 of *Xanthomonas axonopodis* pv. *glycines* can elicit a typical HR in nonhost tobacco [5]; expression of *hpaG*_*Xooc*_ elicits hypersensitive response (HR), which induces disease- and insect-resistance in plants, and enhances plant growth [6]; HrpZ of *Pseudomonas syringae* pv. *phaseolicola* enhances resistance to rhizomania diseases in transgenic *Nicotiana benthamiana* and sugar beet [7].

Intensive studies have revealed that certain regions of harpins are sufficient for eliciting beneficial activities and that each amino acid (aa) residue is critical to the activity. Identification of fragments might facilitate the beneficial application of a harpin-related protein solution to plants in the field and avoid causing negative effects. Mutational analysis of the aa residues between Leu-39 and Leu-50 of HpaG from *Xanthomonas axonopodis* pv. *glycines* determined that the 12 aa residues have crucial roles in HR elicitation in tobacco [8]. Moreover, the HR elicitor activity of the synthetic peptide of this region is the same as that of the HpaG protein at the same concentration [8]; Hairpin XopA did not elicit HR in tobacco. Site directed mutagenesis of Phe48 to Leu48 in XopA resulted in gain of HR function in *X*. *campestris* pv. *vesicatoria* results in the lack of HR-eliciting activity [8]. Both the P44 (aa 269–312) and P24 (aa 290–313) sequences, which represent putative α-helical fragments of HrpZ from *Pseudomonas syringae* pv. *phaseolicola*, induce cell death in tobacco [9]. Interestingly, the domains responsible for eliciting HR reveal that the putative consensus motif has a high level of leucine [9]. In addition, the expression of fragment HpaG_10-42_ from *X*. *oryzae* pv. *oryzicola* not only reduces disease but also increases the yield of rice by promoting plant growth [10–12]. The N-terminus of Hpa1 is a crucial region for promoting leaf photosynthesis by facilitating CO_2_ transport and, hence, plant growth promotion (PGP) [13]. HpaXm of *Xanthomonas citri* subsp. *malvacearum* with two heptads from the N-terminal a-helical region of HpaXm displayed activity in inducing HR [14].

Based on similarity and domain structures, the studied harpins have been categorized into four major groups: the HrpN group, the HrpZ1 group, the HrpW1 group and the Hpa1 group [1]. However, because of its special structure, the novel protein HpaXm of *X*. *citri* subsp. *malvacearum* has not been categorized into one of the four groups [1,14]. Hpa1Xoo of *X*. *oryzae* pv. *oryzae* is a member of the Hpa1 group [1]. To date, few studies have compared the activity of the different harpin groups. Here, in order to determine the function of HpaXm fragments, we used corresponding fragments of Hpa1Xoo as a control.

The synthesized peptide of HpaXm containing two heptads from the N-terminal α-helical region can elicit HR [14]. But whether the two heptads of HpaXm can elicit the SAR and associated defense response like its complete protein has not been reported [14]. This study has just provided the evidence and makes the functions of the fragment more complete. The 12 highly hydrophilic amino acid (aa) residues of Hpa1Xoo, which partially overlap the α-helical region at the N-terminal, are crucial for eliciting HR in a nonhost plant [15]. These studies [14, 15] suggest that the α-helical region may be sufficient for HR. In addition, the synthetic peptides have comparable activity with regard to eliciting HR as that of the expressed protein at the same concentration [8,14]. Moreover, synthetic peptides are readily available materials and can be produced with a high level of purity. Therefore, in this study, we used synthetic peptides of the α-helical fragments, between Ser-35 and Leu-51 (H2N-SEKQLDQLLTQLIMALL-COOH) of HpaXm (HpaXm_35-51_) and between Ser-36 and Leu-52 (H_2_N-SEKQLDQLLCQLISALL-COOH) of Hpa1Xoo (Hpa1Xoo_36-52_), to detect HR elicitor activity. Previous studies have reported that the N-terminal of Hpa1 from *X*. *oryzae* and the fragment of HpaG_10-42_ from *X*. *oryzae* pv. *oryzicola* were both important for promoting plant growth [10, 11, 13]. Therefore, we synthesized peptides of the corresponding domain, between Ala-10 and Leu-39 (H2N-ANSSFLQVDPSQNTQFGPNQGNQGISEKQL-COOH) of HpaXm (HpaXm_10-39_) and between Gly-10 and Leu-40 () of Hpa1Xoo (Hpa1Xoo_10-40_), to detect PGP elicitor activity.

In this study, we demonstrated that α-helical fragments can elicit HR, and that fragments corresponding to HpaG_10-42_ of HpaXm and Hpa1Xoo can induce PGP; furthermore, the fragments still show elicitor activity even when they have been heated. We also determined that although heat stability is a common characteristic of harpins, the degree of heat resistance is different for diverse functional genes associated with the same fragment.

## Materials and methods

### Harpin and peptide preparation

The two strains BL21/pGEX-hpaXm and BL21/pGEX-hpa1Xoo were used in this study were maintained in the lab at -80°C. The two strains were grown in LB medium supplemented with a final concentration of 100μg ml^-1^ ampicillin at 37°C. The two strains were used to prepare protein HpaXm (ACD56757.1) and Hpa1Xoo (ABG36696.1). The protein of HpaXm and Hpa1Xoo with glutathione *S*-transferase (GST) were purified as previously described [14]. Synthetic peptides of the four new fragments HpaXm_35-51_, HpaXm_10-39_, Hpa1Xoo_36-52_ and Hpa1Xoo_10-40_ were synthesized with purities of 96.4%, 97.0%, 94.7%, and 89.1%, respectively, by the GenScript Corporation (Nanjing, China). The synthetic peptides were diluted to 1 mg ml^–1^ in ultrapure water according to the GenScript recommended solvent guidelines and stored at –20°C until ready to use. The two complete harpins and the four new fragments were heated in water for 8 min to determine their heat-stability and were named B-HpaXm, B-Hpa1Xoo, B-HpaXm_35-51_, B-HpaXm_10-39_, B-Hpa1Xoo_36-52_ and B-Hpa1Xoo_10-40_, respectively. The secondary structures, including the α-helical structures of HpaXm and Hpa1Xoo, HpaXm_35-51_ and Hpa1Xoo_36-52_, were predicted by the PSIPRED protein structure prediction program (http://bioinf.cs.ucl.ac.uk/psipred/). The HpaXm and Hpa1Xoo sequences were aligned using the MEGA 7.0 program.

### HR by infiltration

The activity on eliciting HR, in the form of the macro hypersensitive response (macro-HR), was observed by eye as obvious necrosis [11]. The expressed proteins and fragments of HpaXm and Hpa1Xoo and the corresponding heated solutions were diluted from 1 mg ml^–1^ to 10 μM in phosphate buffered solution (PBS). Tests were performed to check the working concentration of each solution needed to elicit HR. The diluted solutions were injected into the leaves of tobacco (*Nicotiana tabacum* cv. Samsun-NN) seedlings (7–8-week-old) using needleless syringes. PBS was injected as the negative control. The macro hypersensitive response on leaves was scored 5 days post injection. Each treatment on 15 plants was repeated three times with similar results.

### Assay for reactive oxygen species

Hydrogen peroxide (H_2_O_2_) is an important signal molecule in the pathway [11, 16, 17], and its accumulation in plant tissues can be detected using 3, 3’-diaminobenzidine (DAB) dyes [18, 19]. The three youngest fully expanded tobacco leaves were evenly sprayed with a 1 μM solution of the unheated or heated protein/synthetic peptide treatments and then harvested 0, 1, 3, 6, 9, 12, 24 and 72 hours post spraying. The H_2_O_2_ concentration of treated leaves was measured using an H_2_O_2_ detection kit (Nanjing Jiancheng Bioengineering Institute, Nanjing, China). Two treated 1.5-cm-diameter leaf samples were homogenized in 1 ml PBS. The homogenate was centrifuged at low speed before combining 0.1 ml of supernatant with reagents supplied in the H_2_O_2_ detection kit. The absorbance of the assay mixture was read at 420 nm. The H_2_O_2_ content was calculated based on a standard curve of known H_2_O_2_ concentration. The H_2_O_2_ accumulation localization in plant can be detected using 3, 3’-diaminobenzidine (DAB) dyes. Treated tobacco leaves were soaked in 1 mg ml^–1^ 3, 3’-diaminobenzidine (DAB) aqueous solution (pH 3.8) at room temperature for 8 h, cleared with ethanol overnight and then observed under the microscope [20]. Each experiment was carried out three times, and each of them was applied to 10 plants in the same way.

### Plant growth promotion assay

Seeds of *Arabidopsis thaliana*, ecotype Columbia, were disinfected in a diluted sodium hypochlorite solution (1.5% (w/v)) for 10 min, followed by centrifugal washing three times and then chilled in ultrapure water at 4°C for 4 days (d). Seeds were soaked in each protein/synthetic peptide solution and the corresponding heated solutions (15 g ml^–1^) for 6 h before placing the seeds on the agar medium or pots [18]. To determine the *Arabidopsis* root growth, treated seedlings were transferred to 10-cm^2^ plates containing MS medium. The plates were placed vertically in 24°C chambers with a 14-h day: 10-h night cycle. Root lengths were observed and measured every 5 days post transference (dpt). To determine the *Arabidopsis* aerial growth, treated seedlings were transferred to 30ml pots containing a one-to-one ratio of vermiculite and nutrient soil. The pots were placed in 24°C chambers with a 14-h day: 10-h night cycle. The effect of each treatment on PGP was assessed by measure the leaf area of the plants after the transfer 30 days using the Image J software. Each experiment was carried out three times, and each of them was applied to 15 seeds.

### qRT-PCR assay

After treating fully expanded leaves with each treatment solution or the corresponding heated solution, quantitative real-time PCR (qRT-PCR) was done to measure the relative transcriptional expression of the HR marker genes *Hsr203J* [21] and *Hin1* [22] (HR markers), the active oxygen metabolism related gene *AOX* [23], the defense related genes *PR-1a*, *PR-2b*, *Chia5*, *NPR1* [17] (defense markers), and the PGP-related gene *NtEXP6* [24] (PGP marker), which were normalized to that of *EF-1a* [23]. qRT-PCR analyses indicated that the expression of *Hsr203J*, *Hin1* (HR markers), and *NtEXP6* (PGP marker) increased with time from 1 hours post treatment (hpt) following treatment with the full-length protein HpaXm. In spite of this, the HpaXm was markedly more effective in inducing expression of *Hsr203J and Hin1* at 6hpt, and inducing expression of *NtEXP6* at 72 hpt (S1 Fig). In this paper, the expression level of HR markers and defense markers were tested at 6 hpt, PGP marker was tested at 72 hpt. Total RNA was isolated using the RNA prep Pure Plant Kit (TIANGEN). Complementary DNA (cDNA) was synthesized using the Fast Quant RT Super Mix Kit (TIANGEN). qRT-PCR assay was performed using SYBR Premix EX Taq kit (TIANGEN). The qRT-PCR primers are shown in Table 1. The qRT-PCR data were reported and calculated based on the normalization gene *EF-1a* using the 2^-ΔΔCT^ method [23]. The following PCR reaction conditions were used: 95°C for 10 min followed by 40 cycles at 95°C for 10 s, 60°C for 35 s, and 72°C for 20 s. 15 plants were tested in each experiment with three replicates.

**Table 1:** Primers used in this study for the amplification of HR markers, defense markers and PGP marker.

Primer	Primer sequence (5’→3’)	PCR product size (bp)
Hsr203J-F	AGCTATGAAAAAGGGGGAAA	253
Hsr203J-R	AACCATTAGAACGTGACAATC	
Hin1-F	TGACTATTAGAAACCCCAACA	234
Hin1-R	CTTCCATCTCATAAACCCCT	
AOX-F	ACAAGGGCAACATTGAGAAC	254
AOX-R	AAAAAGAACATAACAGCGAC	
PR-1a-F	AATATCCCACTCTTGCC	435
PR-1a-R	TATGGACTTTCGCCTCT	
PR-2b-F	CGGCGGGAGCAGTAAAG	141
PR-2b-R	GAACCCTAGCACAACCAAGAC	
Chia5-F	CAGGGCGGCACTGCTTCT	207
Chia5-R	ATTCCATCGCTTCCACTAATA	
NPR1-F	TTCGTCGCTACCGATAACAC	208
NPR1-R	TTCTCGCTGACAAAACGCAC	
NtEXP6-F	CTCAATGGTGTCATGCTGGA	644
NtEXP6-R	GCCGCTTCAGCTCTTCTACA	
EF1a-F	ATCAATCCAGGTCATCATCA	142
EF1a-R	AAGTTCCTTACCAGAACGCC	

### Data analysis

All data were analyzed and evaluated using the Statistical Program for Social Science (SPSS) software. The results were shown as means ± standard deviation (SD) from three replicates. The LSD test was used to determine significant differences in the root length, the leaf area, and gene expressions. The test was used to distinguish differences of mean values between the PBS treatment and unheated/ heated protein/ synthetic peptide treatment, and p<0.05, p<0.01 and p<0.001 were considered to be statistically significant.

## Results

### Analysis of amino acid sequences of specific fragments

**Fig 1:**
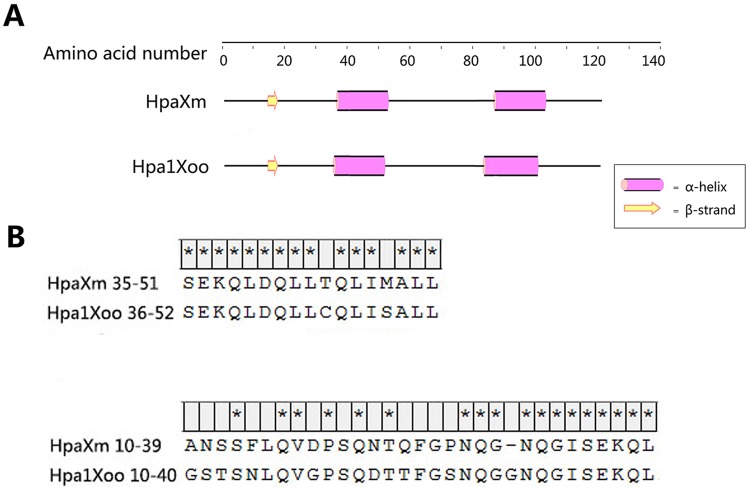
Predicted secondary structures and sequence alignment between HpaXm and Hpa1Xoo. (A) Predicted secondary α-helical and β-strand structures of HpaXm from *Xanthomonas citri* subsp. *malvacearum* and Hpa1Xoo from *Xanthomonas oryzae* pv. *oryzae* using the PSIPRED protein structure prediction program. (B) Sequence alignment of predicted functional domain fragments of HpaXm with the corresponding sequence of Hpa1Xoo. The alignment was produced using the MEGA 7.0 program. Asterisks (*) indicate identical amino acid residues. Alignment of predicted functional domain fragments associated with eliciting the HR (upper). Alignment of predicted functional domain fragments associated with PGP (lower).

Fig 1A shows predicted secondary structures of HpaXm and Hpa1Xoo, which both have one β-strand and two α-helical structures. The HpaXm_35-51_ and Hpa1Xoo_36-52_ fragments contain a α-helix at the N-terminal. Fig 1B shows the sequence alignment of specific fragments of HpaXm and Hpa1Xoo used in this study. HpaXm is a cysteine-free protein, whereas Hpa1Xoo has one cysteine residue at the 45th amino acid site. The homologous position in HpaXm was threonine at the 44th amino acid site (Fig 1). Based on the reported role of the functional domain between Gly-10 and Leu-40 of Hpa1Xoo (Hpa1Xoo_10-40_) in PGP [12], we synthesized the HpaXm_10-39_ peptide to determine its elicitor activity on PGP. The amino acid similarity between HpaXm_10-39_ and Hpa1Xoo_10-40_ was approximately 58.1% and there was a high level of domain similarity from Asn-28 to Leu-40 of HpaXm and from Asn-28 to Leu-39 of Hpa1Xoo.

### Characterization of purified HpaXm and Hpa1Xoo

**Fig 2:**
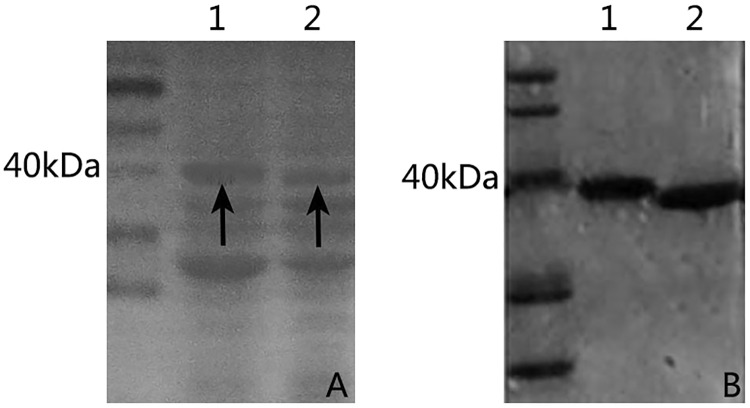
SDS-PAGE analyses of crude/purified harpins GST–Hpa1Xoo (1) and GST–HpaXm (2). The arrows represent the harpins GST–Hpa1Xoo and GST–HpaXm. The crude proteins (A) and the purified proteins (B) were separated by SDS-15% PAGE gel and detected by staining with Coomassie blue.

To analyze the elicitor activity of the four new fragments, HpaXm_35-51_ and Hpa1Xoo_36-52_ on inducing HR, HpaXm_10-39_ and Hpa1Xoo_10-40_ on inducing PGP, we used the corresponding complete proteins HpaXm and Hpa1Xoo as controls. We monitored the complete proteins HpaXm and Hpa1Xoo with glutathione *S*-transferase (GST) using SDS-PAGE after Isopropyl β-D-Thiogalactoside (IPTG) induction training and purification (Fig 2).

### ROS burst and *AOX* gene expression in leaves induced by N-terminal α-helical fragments

**Fig 3:**
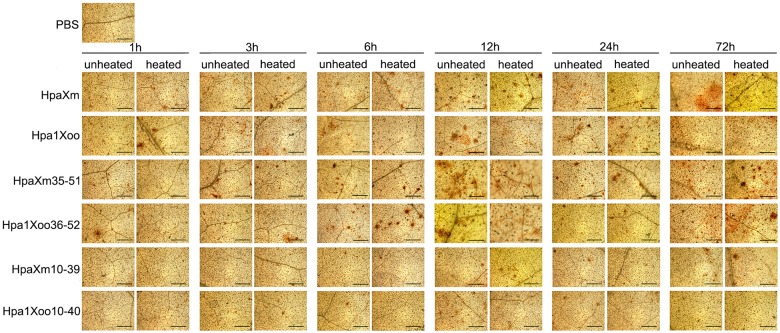
Reactive oxygen species (ROS) burst in tobacco leaves post spraying each solution. Areas that produced a reactive oxygen species (ROS) burst are stained reddish-brown. Scale bars = 1mm. Leaves were infiltrated with the different treatment solutions (15 g ml^–1^) for different periods of time and, then, stained with 1 mg ml^–1^ of DAB solution (pH 3.8) at room temperature for 8 h, cleared with ethanol overnight and then observed under the microscope. A PBS treatment was used as a control. Three leaves on each plant were investigated in the same way.

**Fig 4:**
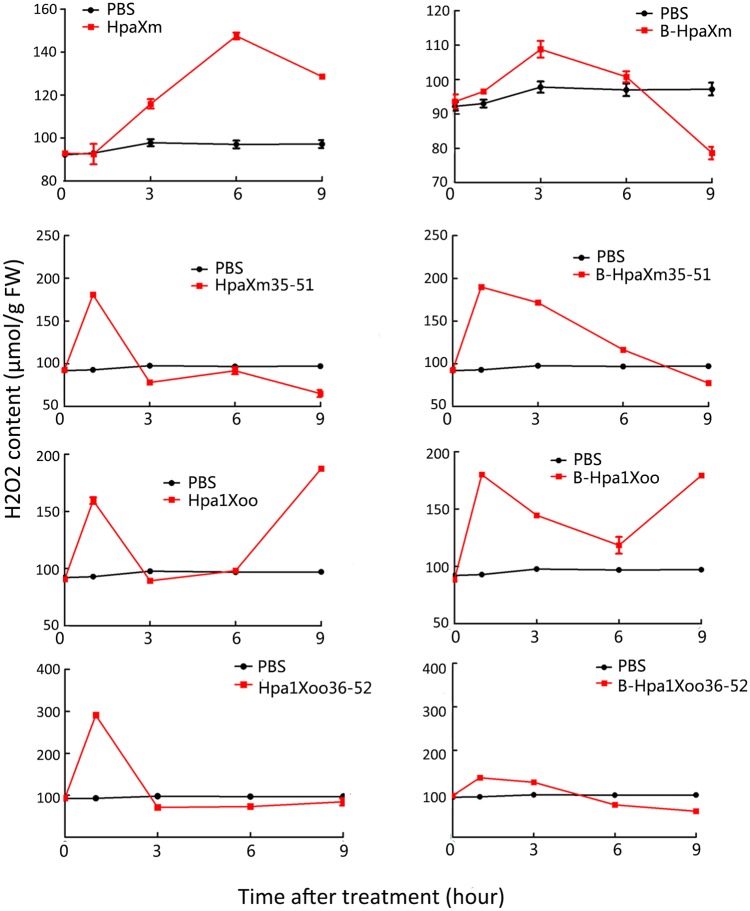
H_2_O_2_ content of tobacco leaves. The H_2_O_2_ content (μmol g^–1^ FW) of tobacco leaves inoculated with 15 μg ml^–1^ of treatment solution; a PBS treatment was used as a control. Bars represent the standard deviation (three replicates).

The presence of reddish or brown spots in tobacco leaves represents the accumulation of H_2_O_2_ and the occurrence of a ROS burst (Fig 3). At 6 hpt, the number of spots was clearly increased in leaves treated with HpaXm_35-51_ or Hpa1Xoo_36-52_, and at 12 hpt in leaves treated with HpaXm or Hpa1Xoo solutions. By contrast, few spots appeared in leaves treated with HpaXm_10-39_ or Hpa1Xoo_10-40_ solutions or PBS. This finding almost corresponds to the H_2_O_2_ content measurements (Fig 4). After treatment with the complete harpin HpaXm, HpaXm_35-51_ or Hpa1Xoo_36-52_, the H_2_O_2_ content of the leaves peaked at 6 h, 1 h, and 1 h, respectively. While for leaves treated with B-HpaXm, B-HpaXm_35-51_ or B-Hpa1Xoo_36-52_, the H_2_O_2_ content peaked at 3 h, 1 h, and 1 h, respectively. After leaves were treated with the complete harpins (Hpa1Xoo or B-Hpa1Xoo), the H_2_O_2_ content increased dramatically and two peaks were observed at 1 h and 9 h. The peak H_2_O_2_ content level in leaves treated with HpaXm solution was 147.5 μmol g^–1^ FW; 180.8 μmol g^–1^ FW in leaves treated with HpaXm_35-51_; 187.4 μmol g^–1^ FW in leaves treated with Hpa1Xoo; and 291.2 μmol g^–1^ FW in leaves treated with Hpa1Xoo_36-52_. The H_2_O_2_ content of leaves treated with PBS was about 95.1 μmol g^–1^ FW. The brown spots representing H_2_O_2_ accumulation obviously increased at 6 hpt / 12 hpt while the H_2_O_2_ quantification data clearly showed H_2_O_2_ peaked at 1hpt / 3hpt. The brown spots were formed with the accumulation of color when DAB, as the chromogenic substrate of preroxidase, react with H_2_O_2_. The presence of brown spots requires a process of color accumulation. Therefore, the H_2_O_2_ peaked before the presence of the brown spots. Moreover, the brown spots can be seen at 1hpt / 3hpt. So the result of the DAB test was consistent with quantification of the H_2_O_2_ concentration. These results show that the ROS burst occurred earlier and stronger when leaves were treated with peptides rather than with complete harpins.

**Fig 5:**
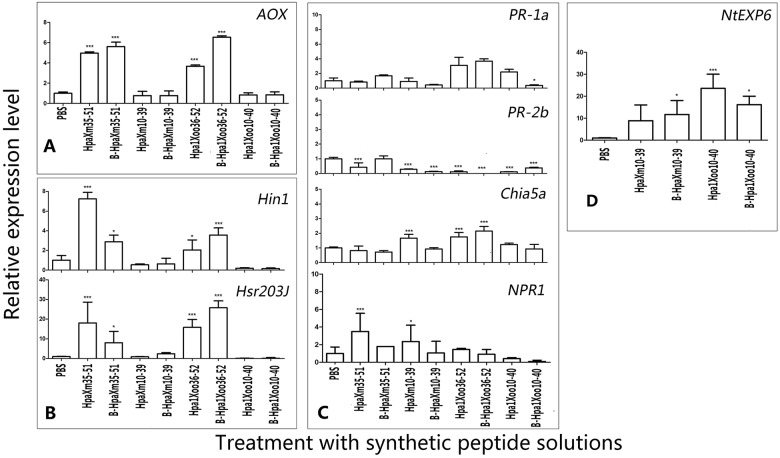
The relative expression levels of active oxygen metabolism-related, HR markers, defense markers, PGP marker in tobacco leaves in response to full-length or synthetic partial fragments of harpins. (A) Relative levels of expression of the active oxygen metabolism-related gene *AOX*. (B) Relative levels of expression of the defense related genes *PR-1a*, *PR-2b*, *Chia5* and *NPR1*. (C) Relative levels of expression of the HR related genes *Hsr203J* and *Hin1*. (D) Relative levels of expression of the PGP related gene *NtEXP6*. In A, B and C, qRT-PCR was performed on RNA isolated at 6 hpt from treated leaves, and in D, on RNA isolated 72hpt. Bars represent the standard deviation (three replicates). Asterisks indicate significant (*P<0.05; **P<0.01; ***P<0.001) differences when compared with the treatment of PBS.

Expression of the *AOX* gene, which is a key gene related to the production of active oxygen, was quantified using real-time (qRT-PCR) (Fig 5A). The *AOX* gene was significantly upregulated (P<0.001) in leaves treated with unheated/heated HpaXm_35-51_ and Hpa1Xoo_36-52_ at 6 hpt. However, the *AOX* gene induced by unheated/heated HpaXm_10-39_ and Hpa1Xoo_10-40_ showed considerable level of expression than it induced by PBS. These results show that the *AOX* gene was activated in leaves induced by N-terminal α-helical fragments. Moreover, the N-terminal α-helical fragments were also heat-stable and show the same degree of elicitor activity to regulate the *AOX* gene at the same concentration.

### HR induced by predicted N-terminal α-helical fragments

**Fig 6:**
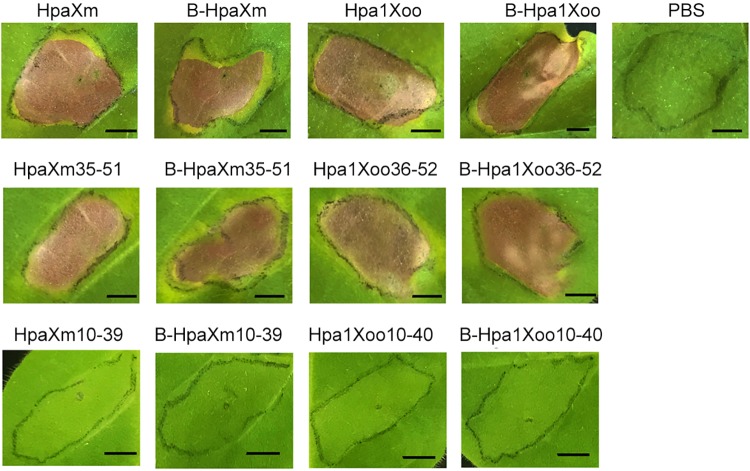
Comparison of the hypersensitive response elicitor activity of the HpaXm and Hpa1Xoo full-length and the synthetic peptides in tobacco leaves five days after infiltration. Tobacco (*Nicotiana tabacum cv*. Samsun) leaves infiltrated with 10 μM samples of HpaXm/Hpa1Xoo unheated or heated protein/synthetic peptide treatments; PBS was used as a control. Scale bars = 1cm.

The activities of HpaXm and Hpa1Xoo and the four new fragments relative to PBS activity were tested in plants. Within 5 d of infiltration into the tobacco leaf intercellular spaces, HpaXm and Hpa1Xoo and HpaXm_35-51_ and Hpa1Xoo_36-52_ induced HR; PBS did not induce HR (Fig 6). Moreover, B-HpaXm, B-Hpa1Xoo, B-HpaXm_35-51_ and B-Hpa1Xoo_36-52_ also induced HR. In other words, the N-terminal α-helical fragments were heat-stable.

To determine the activation of the molecular defense and HR in tobacco plants treated with different solutions, we used qRT-PCR to monitor the mRNA accumulation of these marker genes compared with that of the PBS-treated controls. At 6 hpt, the *Hin1* gene (HR marker) were clearly upregulated (significant difference, P≤0.001) in leaves treated with HpaXm_35-51_ and also upregulated (significant difference, P≤0.05) in leaves treated with Hpa1Xoo_36-52_ (Fig 5B). The *Hsr203J* genes (HR marker) were clearly upregulated (significant difference, P≤0.001) in leaves treated with HpaXm_35-51_ and Hpa1Xoo_36-52_ (Fig 5B). Interestingly, the *Hin1* and *Hsr203J* genes induced by B-Hpa1Xoo_36-52_ showed higher levels of constitutive expression than those induced by Hpa1Xoo_36-52_; however, the genes induced by B-HpaXm_35-51_ showed lower levels of constitutive expression than those induced by HpaXm_35-51_. This result indicates that Hpa1Xoo_36-52_, as a HR elicitor, had stronger activity after heat treatment, whereas HpaXm_35-51_ had weaker HR elicitor activity. This finding shows that the degree of heat tolerance of different harpins within the same functional region is different.

Expression of tobacco genes *PR-1a*, *PR-2b*, *NPR1* and *Chia5a* was tested in response to treatment with synthetic peptide at 6hpt. With the treatment of HpaXm_35-51_, the expression level of *PR-1a*, *PR-2b* and *Chia5a* is lower than the corresponding genes treated with PBS treatment; only the expression level of *NPR1* is higher (significant difference, P≤0.001) than with PBS treatment. With the treatment of Hpa1Xoo_36-52_, the expression level of *PR-1a* and *NPR1* is higher (significant difference, P≤0.001) than with PBS treatment; the expression level of *PR-2b* and *NPR1 is* quite or lower than with the PBS. With the treatment of unheated and heated Hpa1Xoo_36-52_, the defense markers showed considerable level of expression. With the treatment of HpaXm_10-39_ treatment, the genes *Chia5a* and *NPR1* expression level is higher (significant difference, P≤0.001and P≤0.05) than with PBS treatment. With the treatment of B-HpaXm_10-39_, the expression level of defense markers is considerable with the treatment of PBS. With the treatment of Hpa1Xoo_10-40_, the genes *PR-2b*, *Chia5a* and *NPR1* expression level is considerable with PBS treatment; only the *PR-1a* expression level is higher (significant difference, P<0.001) than with PBS treatment. The genes *PR-1a*, *PR-2b*, *Chia5a* and *NPR1* expression level in B- Hpa1Xoo_10-40_ treatment is considerable or lower than in PBS treatment. These results indicate that N-terminal α-helical fragments can induce different defense genes expression in varying degrees, which markedly more active the HR marker genes.

### Effect of specific domain on PGP

**Fig 7:**
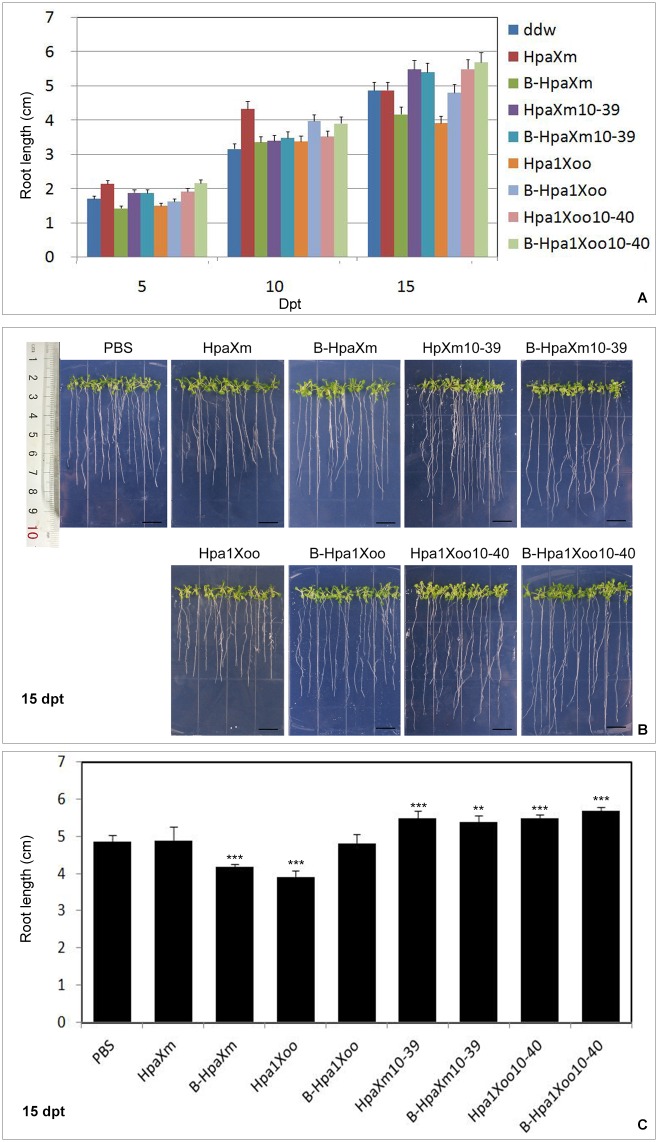
Effects of unheated/heated protein/synthetic peptide on *Arabidopsis* roots growth. (A) Increase in root length with time (roots were measured every 5 days after transfer). The bars indicate the standard deviation (3 replicates). (B) Root growth assay of Arabidopsis seeds grown on MS medium 15 days after transfer. Scale bars = 1cm. (C) Quantification of root growth on agar medium 15 days after transfer. The bars indicate the standard deviation (3 replicates). Asterisks indicate significant (*P<0.05; **P<0.01; ***P<0.001) differences when compared with the treatment of PBS at 15 days.

Fig 7 shows differences in roots length of seeds incubated under vegetable growth conditions among treatments with unheated/heated protein/synthetic peptide solutions (15 g ml^–1^) and PBS. At 10 dpt, roots of plants grown from seeds soaked in each protein/synthetic peptide solution and the corresponding heated solutions were longer than in the PBS (Fig 7A). At 15 dpt, the roots of seeds treated with HpaXm or with B-Hpa1Xoo were as long as that treated with the PBS; the roots of seeds treated with B-HpaXm (significant difference, P<0.001) or with Hpa1Xoo (significant difference, P<0.001) were shorter than that treated with the PBS (Fig 7). At 15 dpt, the roots of seeds treated with unheated/heated synthetic peptide, HpaXm_10-39_ and Hpa1Xoo_10-40_, were longer than with the corresponding unheated/heated completed protein HpaXm and Hpa1Xoo (Fig 7B and 7C). The roots of seeds treated with HpaXm_10-39_(significant difference, P<0.001) or with B-HpaXm_10-39_ (significant difference, P<0.01) were 12.7% or 10.9% longer, respectively, than those treated with PBS; 12.9% or 17.0% with Hpa1Xoo_10-40_ (significant difference, P<0.001) or with B-Hpa1Xoo_10-40_ (significant difference, P<0.001) longer, respectively, than those treated with PBS (Fig 7C).

**Fig 8:**
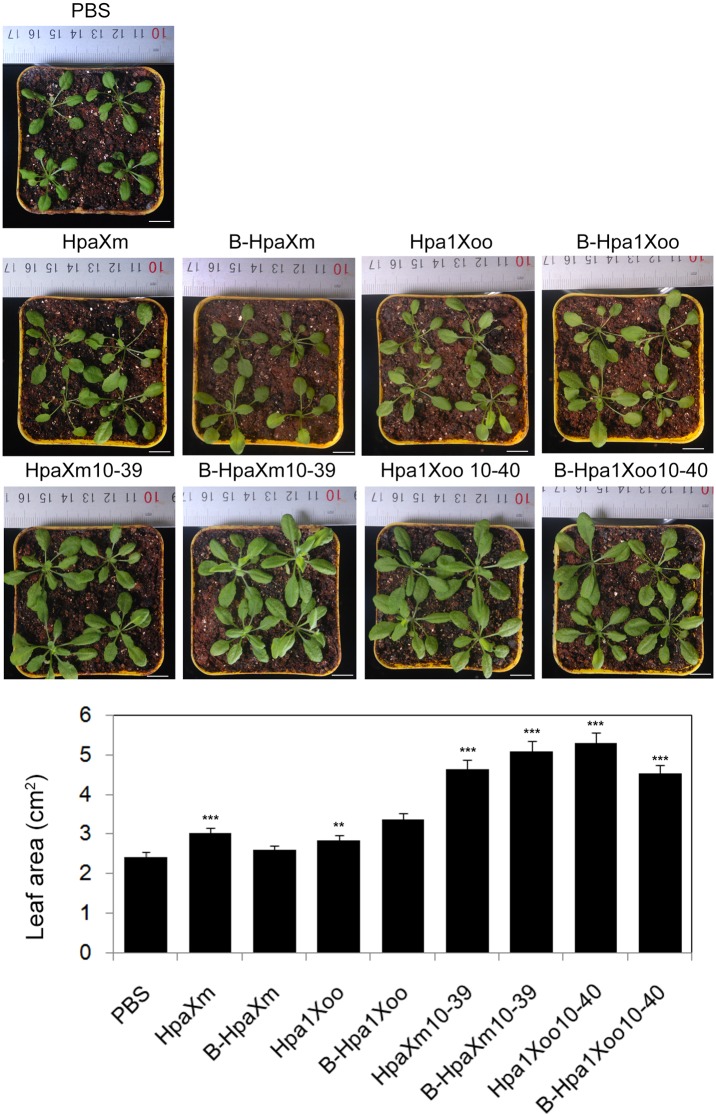
Effects of unheated/heated protein/synthetic peptide on the growth of *Arabidopsis* aerial parts. (A) Appearance of plants grown in pots 30 days after transfer. The seeds were soaked in a 15 μg ml^–1^ solution of unheated heated harpin/synthetic peptide or PBS for 6 h prior to transfer to the pots. Plants were grown in a controlled environment. Scale bars = 1cm. (B) Quantification of plant growth in pots 30 days after transfer. The leaves areas were quantified using the Image J software. Bars represent the standard deviation (three replicates). Black asterisks indicate significant (*P<0.05; **P<0.01; ***P<0.001) differences when compared with the treatment of PBS.

Fig 8 shows differences in aerial parts of plants from seeds treated with unheated/heated protein/synthetic peptide solutions (15 g ml^–1^) and PBS, which were measured by the Image J software. With the treatment of B-HpaXm, the aerial parts of plants were as big as that treated with PBS at 30 dpt (Fig 8A). The leaf area of plants treated with HpaXm were 24.8% larger than that treated with PBS; treatment with Hpa1Xoo were 17.32% larger than that treated with PBS; treatment with B-Hpa1Xoo were 38.8% larger than that treated with PBS (Fig 8B). The aerial parts of plants treated with unheated or heated synthetic peptide treatments, HpaXm_10-39_ and Hpa1Xoo_10-40_, were all clearly larger (significant difference, P<0.001) than those treated with PBS (Fig 8). In addition, the leaf area of plants treated with HpaXm_10-39_ were 92.3% larger than that treated with PBS; treatment with B-HpaXm_10-39_ were 110.9% larger than that treated with PBS; treatment with Hpa1Xoo_10-40_ were 119.5% larger than that treated with PBS; treatment with B-Hpa1Xoo_10-40_ were 87.6% larger than that treated with PBS (Fig 8B). With the treatment of unheated/heated synthetic peptides HpaXm_10-39_ and Hpa1Xoo_10-40_, the aerial parts of plants were larger than those treated with the corresponding unheated/heated complete harpin HpaXm and Hpa1Xoo (Fig 8). It demonstrated that the HpaXm_10-39_ and Hpa1Xoo_10-40_ fragments significantly induced PGP. Moreover, the HpaXm_10-39_ and Hpa1Xoo_10-40_ fragments were heat-stable with different degree.

The *NtEXP6* gene (PGP marker) was upregulated in leaves treated with HpaXm_10-39_ (no significant difference) or Hpa1Xoo_10-40_ (significant difference, P<0.001) at 72 hpt (Fig 5D). Moreover, the *NtEXP6* gene induced by B-HpaXm_10-39_ (significant difference, P<0.05) showed a higher constitutive level of expression than when induced by HpaXm_10-39_. However, the *NtEXP6* gene showed a lower constitutive level of expression when induced by B-Hpa1Xoo_10-40_ (significant difference, P<0.05) than when induced by Hpa1Xoo_10-40_. This result indicates that the HpaXm_10-39_ fragment has stronger activity as a PGP elicitor after heat treatment whereas the HR elicitor activity of B-HpaXm_10-39_ showed the opposite result, indicating that different functional fragments of harpin have different degrees of heat tolerance.

## Discussion

HpaXm is a novel harpin-like protein produced by the cotton leaf blight bacterium *Xanthomonas citri* subsp. *malvacearum*. HpaXm, as an elicitor, can induce HR-mediated disease resistance on the nonhost plant tobacco [14]. In previous studies, the phenotypic experiments of the synthesized N terminal α-domain peptide of HpaXm indicated that the heated/unheated HpaXm_39-52_ can induce HR in non-host tobacco plants [14]. This study not only provides more evidence that the synthetic peptide HpaXm_35-51_ can induce HR, but also provides evidence that another synthetic peptide HpaXm_10-39_ can induce PGP. Interestingly, HpaXm_10-39_ has heat resistance too. Besides, it is the first report to put the unheated/heated complete harpins and the fragments of HpaXm and Hpa1Xoo together to compare their activity on inducing HR or PGP. The synthetic peptides Hpa1Xoo_36-52_ and Hpa1_10-40_ also have heat resistance. Compared with the corresponding fragments of HpaXm, different functional fragments of harpin with the same functional region have different degrees of heat tolerance. Therefore, by determining the elicitor activities of the fragments and the heat tolerance of harpins we should be able to unravel the working mechanisms of harpin and heat resistance of protein, and provide clues for further investigations of the interactions between harpin and the host plant.

Based on similarity and domain structures, the studied harpins have been categorized into four major groups: the HrpN group, the HrpZ1 group, the HrpW1 group and the Hpa1 group [1]. Choi et al. showed that HpaXm as a special novel harpin has not been classified into any of these groups [1]. Based on the description of HpaXm in this paper [1], we propose that HpaXm described in this paper is Hpa2 of *Xanthomonas citri* subsp. *malvacearum* (*Xm*) rather than Hpa1 [14, 25]. However, Hpa1 of *X*. *citri* subsp. *malvacearum* is the Harpin protein that we usually refer to. In the other words, Hpa1 of *Xm*, HpaXm, as a novel harpin has not been categorized to date. Nonetheless, a significant feature of the genes in the Hpa1 group is whether or not their N-terminus has a cysteine residue. HpaXm without a cysteine residue, in a sub-group like HpaG from *X*. *axonopodis* pv. *glycines* and HpaXac from *X*. *axonopodis* pv. *citri*, is distinct from the subgroup containing Hpa1Xoo and Hpa1Xoc [14,26]. The former subgroup contains a threonine residue, but the latter subgroup contains a cysteine in the corresponding position [14]. Moreover, a putative signal peptide (1–15 aa) of HpaXm was predicted in the N-terminal by SignalP (SignalP 3.0 server), which is required for HpaXm to be translocated to the cell wall [27]. A putative signal peptide (1–15 aa) was also found in HpaG and HpaXac, but was not found in Hpa1Xoo and Hpa1Xoc. We propose that the group can be divided into two subgroups, With HpaXm classified in a subgroup with HpaG and HpaXac, and Hpa1Xoo and Hpa1Xoc classified in the other subgroup (unpublished). Thus, because HpaXm and Hp1Xoo appear to belong to different phylogenetic subgroups, we chose Hpa1Xoo to provide a contrast when comparing the activity of Hpa1Xoo with that of HpaXm. The 12 amino acids residues of Hpa1Xoo that partially overlap the α-helical region at the N-terminal are crucial for eliciting HR [15]. Mutation of the N-terminal region of Hpa1Xoo causes the loss of the hypersensitive reaction induction in tobacco [15]. We suggest that the N-terminal α-helix of harpins in the Hpa1 group is the key functional region for HR elicitation. The N-terminal α-helix of HpaXm, like the N-terminal α-helix of Hpa1Xoo, has the ability to elicit HR in tobacco.

A common characteristic of harpins is their heat stability, although more research is needed to understand this mechanism. In this study, some of the genes induced by the heated fragments showed higher levels of upregulation than when induced by the unheated fragments; however, some genes were not induced by the heated fragments. Therefore, exploration of the heat resistance mechanism of harpin and its fragments could provide a theoretical basis for the thermostability of these proteins and help to identify new ways to improve the stability of heat-sensitive proteins. Some explanations for the heat resistance of other thermostable proteins have been reported. First, previous studies had shown that the amino acid composition is closely related to the heat resistance: for example, the heat resistance of thermophilic enzymes with low asparagine (Asn), glutamine (Gln), serine (Ser), or cysteine (Cys) content is related to the deamination of amino acids, β-oxidation, hydrolysis, and the conversion of two disulfide bonds, respectively [28, 29]. However, HpaXm has no Cys and Hpa1Xoo has only one Cys. Perhaps the heat resistance of HpaXm and Hpa1Xoo could be attributed to the lack of obvious tertiary structures connected with disulfide bond formation by cysteine bridges [1]. Second, a thermophilic protein was reported to be a thermostabilization protein that possessed a codon preference for high G and C content at the third position [30]. Interestingly, HpaXm has a codon preference with a high G and C content, but the codon preference of HpaXm is different to that of Hpa1Xoo [14]. Maybe the codon preference is an important heat resistance mechanism and is the reason that HpaXm and Hpa1Xoo have different levels of heat resistance. Finally, the amino acid tyrosine as a target for nitration inhibits carbonic anhydrase activity under high temperature [31]. Interestingly, harpins have low tyrosine content: for example, HpaXm and HpaG only have one tyrosine and Hpa1Xoo and Hpa1Xoc have no tyrosine. We guess the activity of harpins cannot be affected or little affected by the tyrosine nitration under high temperature.

In summary, fragments with different functional domains can elicit different signal activities, such as HR or PGP. However, both the complete harpins and the synthetic peptides of fragments show thermal stability. In the other words, heat stability can be considered a conserved functional characteristic of harpins.

## Supporting information

S1 Fig The relative expression levels of HR marker genes (*Hsr203J* and *Hin1*) and a PGP related gene (*NtEXP6*) in tobacco leaves in response to full-length of HpaXm. The relative expression of the HR marker genes and a PGP related gene in tobacco leaves at different times (0h, 1h, 3h, 6h, 12h, 24h, 72h) after exposure to HpaXm. Relative expression was obtained by normalizing expression to that of *EF-1a* gene. Bars represent the standard deviation (three replicates).
(TIF)
