# Supplementary figures and images for: Functional regions of HpaXm as elicitors with specific heat tolerance induce the hypersensitive response or plant growth promotion in nonhost plants
Source: PLoS One. 2018 Jan 3;13(1):e0188788. doi: 10.1371/journal.pone.0188788 (PMC5751972; doi:10.1371/journal.pone.0188788)

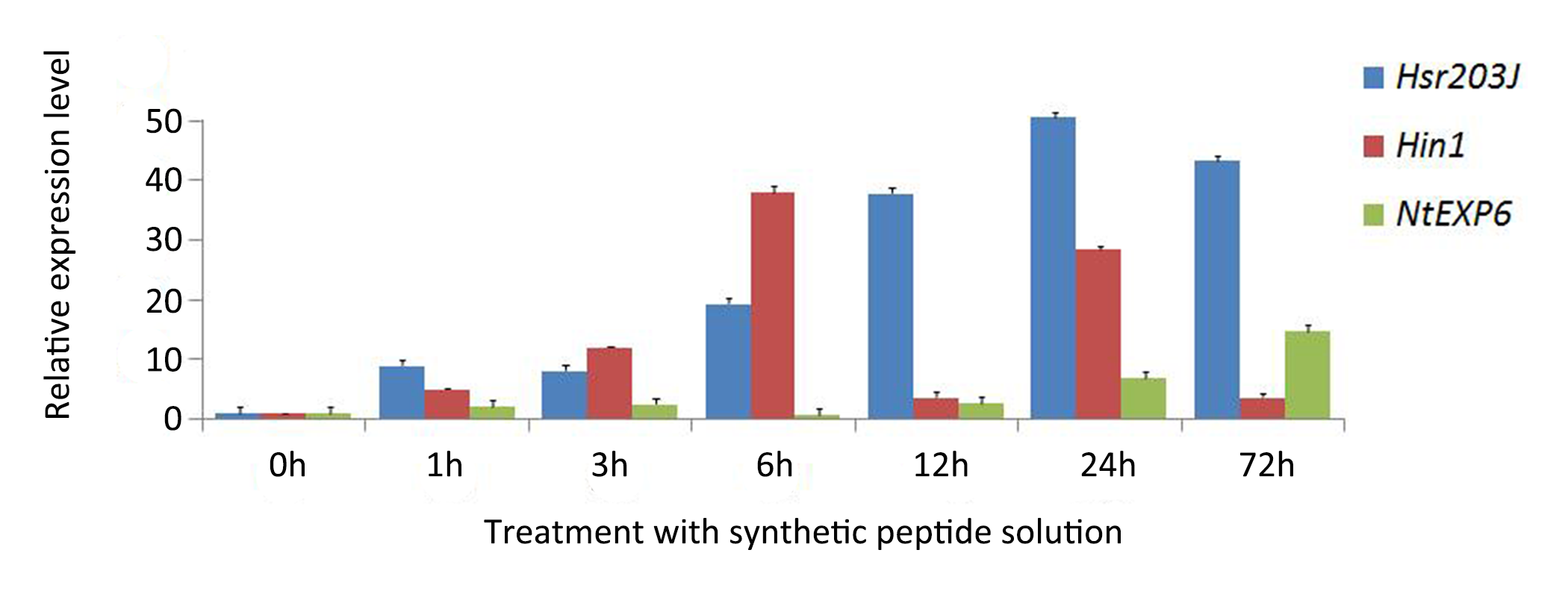

Supplement: S1 Fig — The relative expression of the HR marker genes and a PGP related gene in tobacco leaves at different times (0h, 1h, 3h, 6h, 12h, 24h, 72h) after exposure to HpaXm. Relative expression was obtained by normalizing expression to that of EF-1a gene. Bars represent the standard deviation (three replicates). (TIF) [file pone.0188788.s001.tif]
